# Distinct Roles for Neuropilin1 and Neuropilin2 during Mouse Corneal Innervation

**DOI:** 10.1371/journal.pone.0037175

**Published:** 2012-05-15

**Authors:** Chelsey C. McKenna, Ravi P. Munjaal, Peter Y. Lwigale

**Affiliations:** Department of Biochemistry and Cell Biology, Rice University, Houston, Texas, United States of America; Johns Hopkins University, United States of America

## Abstract

Trigeminal sensory innervation of the cornea is critical for protection and synthesis of neuropeptides required for normal vision. Little is known about axon guidance during mammalian corneal innervation. In contrast to the chick where a pericorneal nerve ring forms via Npn/Sema signaling, mouse corneal axons project directly into the presumptive cornea without initial formation of an analogous nerve ring. Here we show that during development of the mouse cornea, *Npn1* is strongly expressed by the trigeminal ganglion whereas *Npn2* is expressed at low levels. At the same time *Sema3A* and *Sema3F* are expressed in distinct patterns in the ocular tissues. *Npn1^sema−/−^* mutant corneas become precociously and aberrantly innervated by nerve bundles that project further into the corneal stroma. In contrast, stromal innervation was not affected in *Npn2^−/−^* mutants. The corneal epithelium was prematurely innervated in both *Npn1^sema−/−^* and *Npn2^−/−^* mutants. These defects were exacerbated in *Npn1^sema−/−^*;*Npn2^−/−^* double mutants, which in addition showed ectopic innervation of the region between the optic cup and lens vesicle. Collectively, our data show that Sema3A/Npn1 and Sema3F/Npn2 signaling play distinct roles and both are required for proper innervation of the mouse cornea.

## Introduction

The cornea is a transparent, avascular, and highly innervated tissue located at the most anterior part of the eye. It is one of the most innervated tissues of the body, with the nerves providing both sensory information and trophic factors that maintain cornea health and normal vision [1]. Aberrant corneal innervation due to viral infection, trauma or elective surgery can cause neurotrophic keratitis, a condition that leads to degeneration of the cornea [2]. The anatomy and physiology of adult corneal nerves have been very well characterized in many animal models including mouse [3], rats [4], rabbits [5], and humans [6]. These studies show that nerve bundles enter the cornea periphery and radially project towards the center while bifurcating repeatedly into several branches that ultimately innervate the cornea epithelium. Although the development of corneal nerves has been characterized in chick [7], [8] and mouse [9], the molecular mechanisms regulating this process are not well understood.

In the chick embryo, sensory nerves approach the cornea from the ventrotemporal region. These nerves initially avoid the cornea and project dorsally and ventrally forming a ring of nerve fascicles surrounding the developing cornea, also known as the pericorneal nerve ring [7], [8]. In the mouse embryo, nerve bundles approach the cornea from four quadrants situated along the dorsal-ventral and nasal-temporal regions. Unlike corneal innervation in avian embryos, in the mouse, presumptive corneal nerves project directly into the developing cornea without forming a pericorneal nerve ring [9]. Major nerve bundles from all quadrants branch repeatedly as they grow into the corneal stroma and subsequently project anteriorly to innervate the epithelium.

Sensory nerves of the cornea originate from the ophthalmic and maxillary branches of the trigeminal ganglion [10], [11], [12]. Axon projections from the trigeminal ganglion are guided towards their targets by attractive and repulsive signals. Neurotrophins including nerve growth factor (NGF), brain-derived growth factor (BDNF), and neurotrophin-3 (NT-3) play a critical role in attracting sensory nerves to their targets and are expressed by the developing cornea [13]. Conversely, repulsive cues within the craniofacial environment determine their spatiotemporal behavior as they grow towards and innervate their targets. A group of axon guidance molecules known as Semaphorin (Sema), which signal through Neuropilin (Npn) receptors, play a major role in guiding trigeminal axons. Mutant mice lacking proper function of Sema3A or its receptor Npn1 [14], [15], [16], and Sema3F or its receptor Npn2 [17], [18], [19], [20], show severe defasciculation of nerves projecting from the trigeminal ganglion. However, few studies have examined innervation of the target tissues in these mutants at later stages of development.

Several class 3 secreted semaphorins are expressed in the developing chick eye during early development. Of these, *Sema3A* and *Sema3F* are expressed in patterns consistent with a role in cornea development. *Sema3A* is expressed in the lens epithelium, while *Sema3F* is expressed in the adjacent presumptive cornea epithelium [21], [22]. Inhibition of Sema3A signaling in the chick, either pharmacologically or by lens ablation abrogates the formation of the pericorneal nerve ring, which results in precocious and disorganized innervation of the cornea. [8]. Mutant mice lacking Sema3A exhibit misguided axon projections into the eye [15], but have not been studied in detail with respect to corneal innervation. Similarly, the role of Sema3F/Npn2 signaling has not been described during this process. Given that innervation of the mouse cornea is different than chick in that it lacks a pericorneal nerve ring [9], the role of Sema3A/Npn1 and Sema3F/Npn2 signaling in innervation of the mouse cornea remains unclear.

In this study, we investigated the role of Sema3A/Npn1 and Sema3F/Npn2 signaling during mouse corneal innervation. We analyzed the ocular expression of *Sema3A* and *Sema3F*, and their respective receptors *Npn1* and *Npn2* by the trigeminal ganglion over time. To determine the functional significance of Sema3A/Npn1 and Sema3F/Npn2 signaling during mouse corneal innervation, we compared *Npn1^Sema−/−^* and *Npn2^−/−^* mutant corneas with wild type litter mates and observed that mutants were prematurely innervated. Double mutant corneas exhibit increased severity of stromal and epithelial innervation defects as well as ectopic nerve projections between the optic cup and lens vesicle. The results reveal a distinct and important role for Sema3A/Npn1 and Sema3F/Npn2 signaling during innervation of the mouse cornea.

## Materials and Methods

### Mouse embryos

Generation of *Npn1^Sema−/−^*, *Npn2^−/−^*, and double mutant mice lacking Semaphorin signaling through Neuropilins (*Npn1^sema−/−^*;*Npn2^−/−^*) was described previously [18], [23], [24]. Briefly, the *Npn1^Sema−/−^* mice express normal levels of a modified Npn1 protein that binds to VEGF_164_ but due to a 7 amino acid substitution in the binding domain, its interaction with Semaphorin is completely disrupted. *Npn2^−/−^* mice were generated with a complete deletion of the first exon and 1.7 kb of upstream sequence. Animal studies were approved by the Institutional Animal Care and Use Committee (IACUC) at Rice University (approval #A09081401). The mouse lines were maintained through heterozygous matings as separate breeding colonies. Mice with visible vaginal plugs the morning after mating were designated as embryonic day 0.5 (E0.5). For corneal innervation studies, embryos were collected at E12.5–E16.6 in sterile Ringer's solution. Mutant embryos were identified by PCR genotyping using primers for *Npn1^Sema−/−^* [23] and *Npn2^−/−^* [18] with the following modifications to the *Npn2^−/−^* primers: primers used to identify a 400 bp wild type band were (F, 5′-TCAGGACACGAAGTGAGAAG-3′, RV, 5′-GGGAGATGTGTTCTGCTTCA-3′) and primers used to identify 1 Kb mutant band were (F, 5′-CGCATTGCATCAGCCATGAT-3′, RV, 5′-GGGAGATGTGTTCTGCTTCA-3′). PCR reactions were carried out for 35 cycles at 95°C for 1 min, 60°C for 1 min 30 sec, and 72°C for 1 min.

### In situ hybridization

Freshly isolated mouse heads (E12.5–E14.5) or eyes (E16.5) were fixed overnight at 4°C in modified Carnoy's fixative (60% ethanol, 30% formaldehyde, and 10% glacial acetic acid) and embedded in paraffin. At least 3 heads or 3 eyes were analyzed at each developmental stage. In situ hybridization was performed on 10–12 µm sections as described [25]. Mouse digoxigenin-labeled riboprobes for *Npn1* [26], *Sema3A* [27], and *Sema3F* [18] were transcribed as previously described. Mouse *Npn2* riboprobes were synthesized using a TOPO construct carrying a PCR amplified cDNA fragment of Npn2. RNA for making the cDNA was isolated from adult mouse trigeminal ganglia. The following primers were used; F, 5′-GGACTGTACCTTCACCATCCTGGC-3′ and RV 5′-CTGGAAATGTTCTGTCATTGGGGTTAG-3′.

### Immunostaining

Mouse embryos were fixed overnight at 4°C in 4% paraformaldehyde. Whole-mount immunostaining of heads and eyes was performed following standard procedures with minor modifications. Briefly, samples were washed in phosphate buffered saline containing 0.1% Triton-X (PBT) and blocked in PBT containing 0.1% BSA and 5% heat-inactivated sheep serum. Mouse heads between E12.5–E13.5 were incubated overnight at 4°C and eyes from E14.5–E15.5 were incubated overnight at room temperature in antibody solution containing rabbit anti-neuron–specific β-tubulin (TuJ1) IgG antibody (Covance, Richmond, CA) diluted 1∶500 in blocking solution. After extensive washes in PBT, samples were blocked for one hour then incubated overnight at 4°C in secondary antibody (Alexa 594 goat anti-rabbit IgG, Invitrogen, Carlsbad, CA) diluted 1∶200 in blocking solution. Samples were washed and mounted in PBS, then imaged with a Zeiss AxioImager 2 fluorescence microscope with ApoTome and Axiocam (Carl Zeiss AG, Germany). After imaging, samples were embedded in gelatin, cryo-sectioned at 8–10 µm and counterstained with 4,6-diamidino-2-phenylindole (DAPI) to label all nuclei.

### Quantitative analysis of axon lengths

To determine the extent of corneal innervation, a circle was digitally superimposed over the whole mount captured image of the anterior eye to delineate the cornea boundary (300 µm for E13.5, 500 µm for E14.5 and E15.5). At these stages of development, there is no or minimal epithelial innervation and therefore no effort was made to distinguish between stroma and epithelial nerves. Images were processed in ImageJ [28] and nerves were quantified using the NeuronJ plug [29]. The total length of corneal nerves was determined for each cornea. The values reported are averages of each group. Statistical analysis was performed using a One-Way Analysis of Variance (ANOVA) with a Tukey-Kramer Multiple Comparisons Post Test.

## Results

### Spatiotemporal expression of *Npn1* and *Npn2* by the trigeminal ganglion and *Sema3A* and *Sema3F* by ocular tissues during mouse eye development

Sensory innervation of the cornea is derived from the trigeminal ganglion [12], [30], [31]. During chick development, inhibition of Sema3A signaling by the lens disrupts the formation of the pericorneal nerve ring resulting in the mispatterning of corneal nerves [8]. Surprisingly, mice lack a pericorneal nerve ring and instead have direct projection of sensory afferents into the presumptive cornea [9]. Given these species differences in axon patterning, here we examine the role of Npn/Sema signaling in the mouse during corneal innervation.

**Figure 1:**
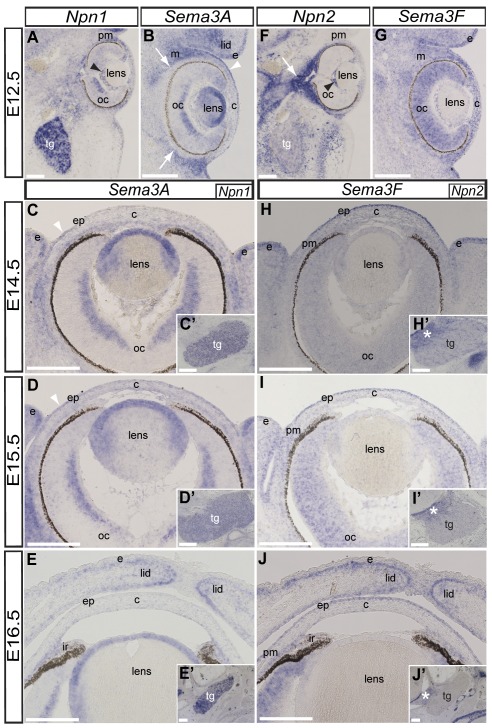
Expression of *Npn1* and *Npn2* and their ligands *Sema3A* and *Sema3F* during eye development. Levels of mRNA expression were revealed by section in situ hybridization on transverse sections through the cranial region of wild-type mice. (A) *Npn1* is expressed in the trigeminal ganglion (tg), periocular mesenchyme (pm), optic cup (oc) and hyaloid vasculature (black arrowhead) at E12.5. (C′–E′) The trigeminal ganglion remains positive for *Npn1* during corneal innervation between E14.5–E16.5. (B) *Sema3A* is expressed in the lens, pericorneal epithelium, and in the ocular and eyelid mesenchyme (m), but void from the periocular region (arrows) at E12.5. (B–E) This pattern of *Sema3A* expression is maintained at E14.5–E16.5, except in the pericorneal epithelium (white arrowheads) where it is down regulated at E16.5. (F) In contrast with *Npn1*, *Npn2* is expressed at low levels in the trigeminal ganglion but strongly expressed around the optic nerve (arrow), hyaloid vasculature, and periocular mesenchyme at E12.5. (H′–J′) Low levels of Npn2 expression are maintained in the trigeminal ganglion throughout E14.5–E16.5 with strong expression restricted to the trigeminal root (asterisks). (G) Expression of *Sema3F* is ubiquitous in the eye region at E12.5. (H–J) Between E14.5–16.5, expression of *Sema3F* is prominent in the corneal epithelium, optic cup, and eyelids. Scale bars: 200 µm.

As an initial step, we established the expression of *Sema3A* and *Sema3F*, and their respective receptors *Npn1* and *Npn2* during eye development. Embryos were analyzed between E12.5 and E16.5, a period corresponding to the time when presumptive corneal nerves first appear at the periphery of the anterior eye region until when the corneal stroma and epithelium are innervated [9]. The expression patterns of these mRNAs at E13.5 (data not shown) are similar to E12.5 (Fig. 1A, 1B, 1F and 1G).

### Expression pattern of *Npn1* and *Sema3A* during mouse corneal innervation

In situ hybridization on sections through the craniofacial region at E12.5 shows strong expression of *Npn1* mRNA by the trigeminal ganglion, central region of the optic cup, hyaloid vasculature, and periocular mesenchyme (Fig. 1A). Strong expression of *Npn1* persists in the trigeminal ganglion throughout the period of corneal innervation (Figs. 1C′, 1D′, and 1E′), consistent with previous observations [14], [32].

At E12.5, expression of *Sema3A* mRNA is prominent in several ocular tissues including the lens vesicle, central region of the optic cup, in the presumptive eyelid ectoderm (Fig. 1B), and pericorneal epithelium (Fig. 1B; arrowhead). Although *Sema3A* is expressed in the ocular mesenchyme and eyelid, it is absent from the region adjacent to the optic cup (Fig. 1B; arrows) along which trigeminal nerves project towards the presumptive cornea [9]. Apart from the pericorneal epithelium (arrowheads) and eyelid ectoderm where *Sema3A* is down regulated, its expression pattern persists in the ocular tissues up to E16.5 (Figs. 1C, 1D, and 1E). *Sema3A* expression becomes prominent in the presumptive iris and iridocorneal angle (region between the cornea and iris) at E16.5 (Fig. 1E). Our results show that, similar to chick [8], mouse *Npn1* is expressed in the trigeminal ganglion and *Sema3A* is expressed in ocular tissues in patterns consistent with a possible role of Npn1/Sema3A signaling in guiding trigeminal afferents during corneal innervation.

### Expression pattern of Npn2 and Sema3F during mouse corneal innervation

To determine whether Npn2/Sema3F, like Npn1/Sema3A, are candidates for mediating axonal guidance during mouse corneal innervation, we examined the expression of *Npn2* and *Sema3F* mRNA between E12.5 and E16.5 by in situ hybridization in tissue sections. Previous studies have shown that *Npn2* is expressed by the trigeminal ganglion during gangliogenesis between E9 and E11.5 [17], [18], [20] and overlaps with *Npn1* expression at these stages [14], [32]. Interestingly, we found that *Npn2* is expressed at relatively low levels in the trigeminal ganglion at E12.5 (Fig. 1F), compared to *Npn1* expression at the same stage (Fig. 1A). However, similar to *Npn1*, *Npn2* is expressed in the periocular mesenchyme and hyaloid vasculature. *Npn2* is also strongly expressed in the mesenchyme around the optic nerve (Fig. 1F, arrow). Between E12.5 and E16.5, expression of *Npn2* remained low in the trigeminal ganglion but high expression persisted in the trigeminal nerve root where it attaches to the central nervous system (Figs. 1H, 1I, and 1J; asterisk).

*Sema3F* is ubiquitously expressed at low levels in the ocular and frontal-nasal regions of the mouse between E9.5 and E11 [18], [20], [33]. As previously shown, we found that at E12.5, *Sema3F* is ubiquitously expressed at low levels in the presumptive cornea, optic cup, and ocular mesenchyme (Fig. 1G). Between E14.5 and E15.5 expression of *Sema3F* remains at low levels in the eyelid mesenchyme, optic cup, periocular mesenchyme and presumptive corneal stroma, but becomes prominent in the eyelid and corneal epithelium (Figs. 1H and 1I). By E16, expression of *Sema3F* persists in the corneal epithelium and becomes strong in presumptive iris and iridocorneal angle, optic cup, and in the eyelid mesenchyme adjacent to the ectoderm (Fig. 1J). The low levels of *Npn2* expression by the trigeminal ganglion coupled with low levels of *Sema3F* expression by the ocular mesenchyme and cornea indicate that Npn2/Sema3F signaling may play a minor role in guiding axons during their projection into the corneal stroma. However, high levels of *Sema3F* expression in the cornea epithelium at E16.5 may play a role during epithelial innervation.

### Npn1 but not Npn2 regulates innervation of the mouse corneal stroma

**Figure 2:**
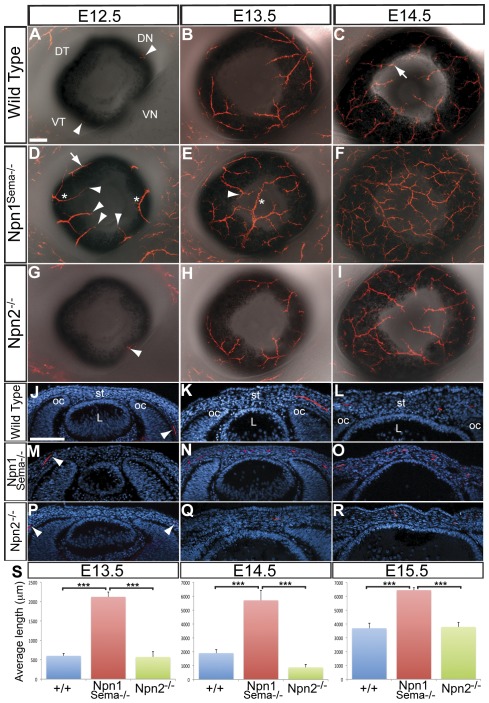
Innervation of the corneal stroma is defective in *Npn1^Sema−/−^* but normal in *Npn2^−/−^* mutant embryos. Immunostaining for TuJ1 was performed on whole-mount corneas (A–I) and sections (J–R) at E12.5, E13.5 and E14.5. (A–C) Innervation of wild type cornea showing the spatiotemporal projection of nerve bundles into the four quadrants of the stroma and minimal contribution from the DT quadrant (arrow in C). (D) At E12.5, *Npn1^Sema−/−^* eyes show several aberrant projections (asterisks) that also innervate the DT quadrant (arrow) and extend close to the corneal periphery (black arrowheads). (E) By E13.5, nerve bundles from all quadrants including the DT quadrant (arrowhead) innervate the cornea and some project across the diameter of the cornea (asterisk). (F) At E14.5, extensively bifurcated nerve bundles cover most of the cornea. (G–I) Innervation of *Npn2^−/−^* eyes resembled wild type at these stages of corneal development. (J–R) Representative sections through wild type, *Npn1^Sema−/−^* and *Npn2^−/−^* mutant corneas showing the extent of nerve projections at each developmental stage. All sections were counterstained with DAPI. Arrowheads in J, M, and P show relative projection of nerve bundles in the periocular region at E12.5. (S) Quantification of nerves in wild type, *Npn1^Sema−/−^* and *Npn2^−/−^* mutant corneas was carried out by measuring the lengths of nerve bundles in the cornea at E13.5–E15.5; for details see Methods section. For all samples n = 8, except *Npn2^−/−^* mutants where n = 6 for E14.5 and E15.5. Error bars = SEM. Scale bars: 100 µm. ***, P<0.001.

Mouse embryos lacking either Npn1 [14], [23], [34], [35] or Npn2 [18], [20], exhibit defects in trigeminal ganglion development including severe defasciculation of nerve projections. Similar trigeminal defects are observed in mice lacking Sema3A [16] or Sema3F [20] function. Given that *Sema3A* and *Sema3F* are expressed in ocular tissues in the pathway of trigeminal sensory projections, we asked whether Npn1 and Npn2 are required for nerve guidance during mouse corneal innervation. Since Npn1^−/−^-null mutant mice die at E8.5 from vascular defects due to lack of signaling from vascular endothelial growth factor [36], we used a line of Npn1 mutant mice that is only defective in Npn1/Sema signaling [23], [24] and is viable during the period of corneal innervation. Nerve projections were examined in wild type, *Npn1^Sema−/−^*, and *Npn2^−/−^* mutant embryos that were stained with the TuJ1 antibody to identify axonal projections. Previously, we showed that in wild type mice, presumptive corneal nerves first appear in the anterior region of the eye at about E12.5 [9]. At this stage, anterior nerve projections are absent in most wild type eyes (n = 24/35), but a few embryos show initial nerve projections in the dorsal-nasal (DN), ventral-nasal (VN), or the ventral-temporal (VT) quadrants (n = 11/35; Figs. 2A and J). *Npn1^Sema−/−^* eyes (n = 6) are innervated in all quadrants including the DT quadrant (Fig. 2D; arrow). The DT quadrant is never innervated at this time in wild type eyes. *Npn1^Sema−/−^* eyes display ectopic nerve bundles (Fig. 2D; asterisks) projecting between the entry points of the four major nerve bundles which are found in wild type eyes, and subsequently innervate the entire corneal stroma [9]. In addition, the nerve bundles project into the corneal periphery of E12.5 *Npn1^Sema−/−^* eyes (Figs. 2D and M; arrowheads), which normally occurs later at about E13.5 in wild type (Fig. 2B). In marked contrast to *Npn1^Sema−/−^*, axon projections into *Npn2* ^−/−^ eyes (n = 8) were apparently not affected and resembled wild type at E12.5 (Figs. 2G and 2P). By E13.5, axons from the DN, VN, and VT major nerve bundles extend as far as the corneal periphery and the DT quadrant is minimally innervated in some of the wild type eyes (n = 15, Fig. 2B). At this stage in *Npn1^Sema−/−^* eyes (n = 8), nerve bundles continue to project from all quadrants and innervate the stroma (Figs. 2E and N). Nerve bundles in the DT quadrant projected further and innervated the stroma of *Npn1^Sema−/−^* corneas (Fig. 2E; arrowhead). In some cases nerve bundles grew past the corneal center and projected across its diameter (Fig. 2E; asterisk). *Npn2* ^−/−^ eyes (n = 6) showed similar patterns of innervation as wild type at this stage (Figs. 2H and 2Q). By E14.5, major nerve bundles from the DN, VN, and VT quadrants innervate the stoma and project towards the center (Figs. 2C and 2L). The major nerve bundle in the DT quadrant extends a relatively short distance compared to nerve bundles in the other quadrants, and at this time it innervates the corneal periphery (Fig. 2C; arrow). At E14.5, *Npn1^Sema−/−^* corneas are innervated by numerous axons that repeatedly bifurcate from the major nerve bundles and project throughout the stroma (Figs. 2F and O). Innervation of *Npn2* ^−/−^ corneas (n = 6) appears similar to wild type (Figs. 2I and 2R).

Because of variations in the extent of axon projections into the cornea within wild type and mutant embryos, we quantified axon lengths within the cornea during stromal innervation (Fig. 2S). We found that axons grew significantly further in *Npn1^Sema−/−^* corneas at E13.5 (n = 8; p<0.0001), E14.5 (n = 8; p = 0.0007), and E15.5 (n = 8; p = 0.0133) compared to wild type corneas. *Npn2* ^−/−^ corneas did not show significant difference in axon length at E13.5 (n = 6; p = 0.8833), E14.5 (n = 6; p = 0.0455) or E15.5 (n = 8; p = 0.7168) relative to wild type. Altogether, our results indicate that Npn1 is required for axon guidance during the innervation of the mouse corneal stroma but Npn2 plays an insignificant role during this process.

### Innervation of the corneal quadrants in *Npn1^Sema−/−^* and *Npn2* ^−/−^ mutant mice

As shown above and previously described [9], a unique characteristic of normal mouse corneal innervation is that all nerves originate from four major nerve bundles that repeatedly branch as they innervate the corneal stroma and epithelium. Although the major nerve bundles from the DN, VN, and VT quadrants project into the cornea at the same rate and innervate approximately similar surface areas, the DT quadrant grows relatively slowly and therefore covers a smaller area. To further determine if there were aberrant nerve projections in *Npn1^Sema−/−^* and *Npn2^−/−^* mouse corneas, we quantified the extent of stromal innervation in each quadrant by measuring the total length of axons using the NeuronJ plug-in for the ImageJ software. Statistical analysis was performed on measurements taken from wild type, *Npn1^Sema−/−^* and *Npn2^−/−^* corneas at E13.5–E15.5.

**Figure 3:**
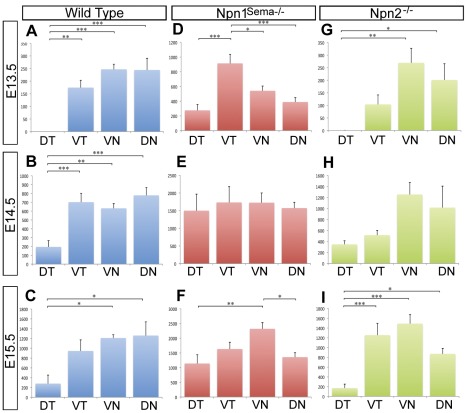
Loss of Npn1 signaling through Sema disrupts the pattern of innervation of the corneal quadrants. Whole mount corneas were immunostained with TuJ1 and imaged. The lengths of nerve bundles projecting into each quadrant were quantified as described in the Methods section. For all graphs, Y-axis is average length of corneal nerves (µm) and X-axis is cornea quadrants. (A–C) Wild type, (D–F) *Npn1^Sema−/−^* mutant, and (G–I) *Npn2^−/−^* mutant eyes were analyzed at E13.5–15.5. ANOVA with a Tukey post test was performed on all data sets. For all samples n = 8, except *Npn2^−/−^* mutants where n = 6 for E14.5 and E15.5. No bracket indicates P>0.05; *, P<0.05; **, P<0.01; ***, P<0.001.

During the onset of stromal innervation in wild type E13.5 (n = 8) and at E14.5 (n = 8) corneas, we observed a significant bias of axons away from the DT quadrant compared to other quadrants (Figs. 3A and 3B). By E15.5 (n = 8), nerves in the DT quadrant project further towards the cornea center (Fig. 2M). Although there are relatively fewer nerve bundles in the DT quadrant at this time, we observed no difference in the innervation of the DT and VT quadrants. Nonetheless the DT quadrant remained less innervated than the VN and DN quadrants (P<0.05; Fig. 3C). Conversely, stromal innervation of *Npn1^Sema−/−^* corneas at E13.5 (n = 8) showed no bias away from the DT quadrant and the VT quadrant was more innervated (P = 0.001; Fig. 3D). By E14.5 (n = 8) all quadrants in *Npn1^Sema−/−^* corneas were equally innervated (P<0.05; Fig. 3E). At E15.5 (n = 8), the VN and DN quadrants were innervated at about the same level (P<0.05) but more than the DT and VT quadrants (P = 0.001; Fig. 3F). At these stages of development, innervation of *Npn2^−/−^* stromal quadrants was similar to wild type (compare Figs. 3G–3I and 3A–3C). Our results show random projection of nerve bundles during the innervation of *Npn1^Sema−/−^* corneal stroma compared to *Npn2^−/−^* and wild type, further suggesting the requirement of Npn1 but not Npn2 during stromal innervation.

### Npn1 and Npn2 play a role during innervation of the corneal epithelium

**Figure 4:**
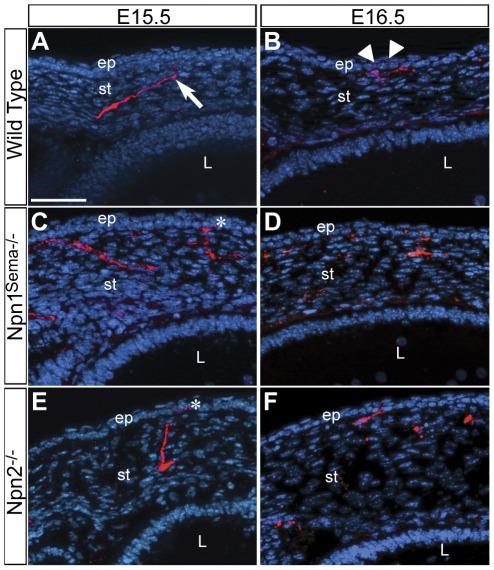
Timing of the innervation of the corneal epithelium is disrupted in *Npn1^Sema−/−^* and *Npn2^−/−^* mutants. Cross sections of E15.5–E16.5 (A–B) Wild type, (C–D) *Npn1^Sema−/−^*, and (E–F) *Npn2^−/−^* corneas immunostained with TuJ1 and counterstained with DAPI. In wild type corneas, axons projection toward the epithelium at E15.5 (A) and innervate it at E16.5 (B). The corneal epithelium is innervated by E15.5 in *Npn1^Sema−/−^* (C, asterisk) and *Npn2^−/−^* (E, asterisk) mutant corneas. Scale bar: 50 µm.

As nerve bundles grow toward the central region of the cornea, both large and small branches project anteriorly toward the epithelial layer at E15.5 (Fig. 4A, arrow), subsequently innervating it at E16.5 (Fig. 4B, arrowheads). This change in the direction of axon projections during stromal innervation is probably in response to the repulsive Sema3A cues emanating from the lens. In addition, expression of *Sema3F* is prominent in the epithelial layer at these stages (Fig. 1H, 1I and 1J) and probably plays a role during its innervation. To determine whether Npn1 and Npn2 play a role during innervation of the corneal epithelium, we analyzed corneal sections from E15.5 and E16.5 *Npn1^Sema−/−^* and *Npn2^−/−^* mutant embryos. The first evidence of epithelial innervation in *Npn1^Sema−/−^* (Fig. 4C, asterisk; n = 5/7) and *Npn2^−/−^* (Fig. 4E, asterisk; n = 4/7) corneas was at E15.5, a day earlier than wild type (n = 0/13). By E16.5, there was continued innervation of the epithelium in both *Npn1^Sema−/−^* and *Npn2^−/−^* corneas (Fig. 4D and 4F; n = 5/5 each) without apparent difference in the number of fascicles compared to wild type (n = 11/11). Thus both Npn1 and Npn2 are required for the proper timing of epithelial innervation.

### Corneal innervation defects are enhanced in *Npn1^Sema−/−^;Npn2^−/−^* double mutants

**Figure 5:**
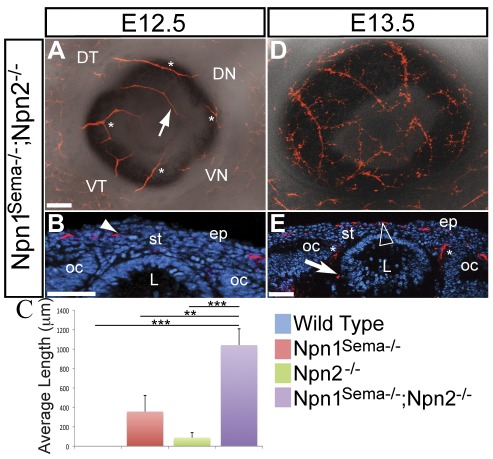
Increased disruption of corneal innervation in *Npn1^Sema−/−^;Npn2^−/−^* double mutants. Whole mount and sections of E12.5–E13.5 eyes were immunostained with TuJ1. Sections were counter stained with DAPI. (A–B) Several nerve bundles projected into ectopic regions of the anterior eye (asterisks), the dorsal temporal region (arrow) and presumptive stroma (arrowhead) are prematurely innervated. (C) Quantification of axon lengths at E12.5 showed that more nerve bundles innervated the *Npn1^Sema−/−^;Npn2^−/−^* double mutant cornea than *Npn1^Sema−/−^* or *Npn2^−/−^* alone. The quantification method is described in the Methods section. No bracket indicates a relationship of P>0.05; **, P<0.01; ***, P<0.001. (D–E) By E13.5, several nerve bundles project towards the central cornea. The presumptive corneal epithelium is innervated (arrowhead), and some nerve bundles project into ectopic regions near the lens vesicle (arrow) and between the lens and optic cup (asterisks). Scale bar: 100 µm. oc, optic cup; c, cornea.

Double mutant mouse embryos lacking semaphorin signaling through Npn1 and Npn2 (*Npn1^Sema−/−^;Npn2^−/^* ^−^) show increased disorganization of cranial ganglia including the trigeminal ganglion compared to *Npn1^Sema−/−^* and *Npn2^−/−^* single mutants [37]. Since corneal sensory nerves are derived from the trigeminal ganglion [12], we asked whether the synergistic defects observed in the trigeminal ganglion of double mutants were recapitulated during corneal innervation. We found that similar to *Npn1^Sema−/−^* (Fig. 2B), several axons projected into the anterior eye region of E12.5 *Npn^Sema−/−^;Npn2^−/−^* double mutant embryos (Fig. 5A). Some axons projected into ectopic regions (Fig. 5A; asterisks) and the DT quadrant was innervated (Fig. 5A; arrow). Cross-sections through E12.5 double mutant eyes revealed that axons projected into the presumptive corneal stroma (Fig. 5B; arrowhead). Comparisons of nerve lengths revealed that *Npn1^Sema−/−^;Npn2^−/−^* double mutant eyes (n = 4) were more innervated than *Npn1^Sema−/−^* (p<0.01) and *Npn2^−/−^* (p<0.001) single mutants (Fig. 5C). At E13.5, we observed that defects in corneal innervation were further enhanced in double mutants. The double mutant cornea was innervated by several axons that projected towards the center (Fig. 5D). Due to difficulties in obtaining *Npn1^Sema−/−^;Npn2^−/−^* embryos (1/16 from an average litter of 8 embryos), we were unable to quantify the defects in axon lengths. However, transverse sections through an E13.5 *Npn1^Sema−/−^;Npn2^−/−^* cornea indicate that the nerve bundles not only project through the presumptive stroma, but also prematurely innervate the corneal epithelium (Fig. 5E, arrowhead). In addition, we observed ectopic nerve projections near the lens vesicle (Fig. 5E, arrow) and between the optic cup and lens vesicle (Fig. 5E, asterisks). Although aberrant innervation of the lens vesicle was previously reported in *Sema3A* ^−/−^ mutants [15], it was not evident in the *Npn1^sema−/−^* (Fig. 2N), *Npn2^−/−^* (Fig. 2Q), or wild type (Fig. 2K) eyes at E13.5. Our results further suggest that both Npn1 and Npn2 are required for and play distinct roles during mouse corneal innervation.

## Discussion

Sensory nerves originating from the trigeminal ganglion innervate the cornea and play a major role in maintaining its homeostasis and transparency. Although Sema/Npn signaling is critical to the formation of the trigeminal ganglion [14], [15], [20], [23], [37], very little is known about its role during mouse corneal innervation. Previous studies in chick have shown that Sema3A/Npn1 signaling regulates the formation of the pericorneal nerve ring that is crucial for the proper patterning of corneal nerves [8], [38]. However, the mouse cornea develops differently, such that nerve bundles project directly into the presumptive cornea without initial formation of a pericorneal nerve ring [9].

**Figure 6:**
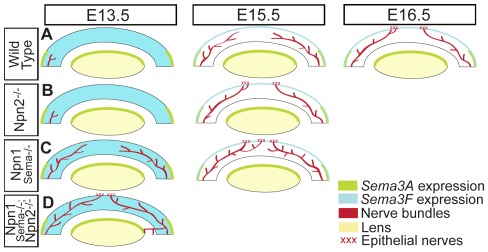
Proposed model for Sema/Npn signaling during mouse corneal innervation. (A) Schematic diagram showing the spatiotemporal expression of *Sema3A* and *Sema3F* in wild type embryos, which guide axons during stromal and epithelial innervation. Innervation of the wild type mouse corneal stroma and epithelium respectively commence at E13.5 and E16.5. (B) In *Npn2^−/−^* mutant embryos, stromal innervation is similar to wild type but the epithelium is prematurely innervated by E15.5. (C) In *Npn1^Sema−/−^* mutants, the stroma and epithelium are both prematurely innervated by several nerve bundles that are misguided into the cornea. (D) The corneal innervation defects shown in *Npn1^Sema−/−^* and *Npn2^−/−^* mutants are exacerbated in the absence of Sema/Npn signaling (*Npn1^Sema−/−^;Npn2^−/−^* double mutants) by E13.5. In addition, nerve bundles project into ectopic regions of the eye between the optic cup and lens vesicle.

This led us to investigate whether Npn1/Sema3A and Npn2/Sema3F signaling play a role in guiding trigeminal afferents during mouse corneal innervation. Our results show that Sema3A/Npn1 is required for proper innervation of the corneal stroma and epithelium, whereas Sema3F/Npn2 is only required during epithelial innervation. Thus Sema3A/Npn1 and Sema3F/Npn2 are both required and play distinct roles during mouse corneal innervation.

Several studies have shown that both *Npn1* and *Npn2* are expressed in the trigeminal ganglion during early development of the mouse embryo [20], [39]. However these studies only addressed trigeminal expression of *Npn1* and *Npn2* during gangliogenesis up to E12.5. Furthermore, the expression of *Sema3A* and *Sema3F* during mouse ocular development was previously unknown. Since trigeminal afferent axons are guided by signals from their targets and surrounding tissues [8], [40], [41], we first established the expression of *Npn1* and *Npn2* in the trigeminal ganglion, and the ocular expression of *Sema3A* and *Sema3F* during corneal innervation between E12.5–E16.5. We found that *Npn1* is expressed prominently and continuously in the trigeminal ganglion. This pattern of expression is conserved between chick [8] and mouse. Suggesting that in addition to guiding neuron progenitor cells during gangliogenesis, Npn1 plays an important role in guiding axon projections towards their targets. Our results also show that *Sema3A* is abundantly expressed in the lens but absent from the mesenchyme adjacent to the optic cup through which axons traverse as they project towards the cornea [9]. During chick eye development, *Sema3A* is expressed in the lens vesicle, presumptive corneal epithelium, and mesenchyme of the presumptive iris and ciliary body [21], [22], [38]. In vitro, Sema3A from the lens repels trigeminal axons whereas its inhibition in vivo results in precocious innervation of the cornea [8]. Therefore the conserved expression of *Sema3A* in the mouse lens suggests that it plays a similar role of guiding trigeminal axons during mouse corneal innervation. Unlike chick, Sema3A signaling by the mouse lens does not lead to the formation of a pericorneal nerve ring. One possibility is that differences between chick and mouse cornea development may play a part in the magnitude of lens-derived Sema3A signal the nerve bundles receive as they approach the eye. In chick, trigeminal afferents reach the pericorneal region at about E4.5 as the first wave of neural crest cells form the corneal endothelium and are repulsed dorsally and ventrally [7], [8]. This contrasts with the mouse where axons reach the corneal periphery at about E13.5, subsequent to the migration of numerous neural crest cells between the lens and corneal epithelium [42], which may buffer the Sema3A secreted by the lens. Alternatively, a combination of Sema3A and other guidance signals in the periocular region may be required for pericorneal nerve ring formation since inhibition of Sema3A during chick corneal innervation does not completely abolish the pericorneal nerve ring [8].

The trigeminal ganglion expresses *Npn2* at about E9.5 where it plays a role during gangliogenesis [20]. Our results show low levels of *Npn2* expression in the trigeminal ganglion by E12.5 and this level of expression is maintained during corneal innervation, except for the strong expression that is restricted to the trigeminal root where it attaches to the central nervous system. Expression of *Sema3F* is diffuse in the ocular tissues at E12.5 but becomes restricted to the corneal epithelium at E14–E16.5. Similarly, expression of *Sema3F* is prominent in the presumptive corneal epithelium of the chick [21], but was not examined at later stages of corneal development. The low levels of *Npn2* expression in the trigeminal ganglion coupled with the low expression of *Sema3F* in the corneal stroma suggest that Sema3F/Npn2 signaling is dispensable during innervation of the corneal stroma. However, the relatively high expression of *Sema3F* in the corneal epithelium may suffice in guiding axons during epithelial innervation.

Given that *Npn1* and *Npn2* are expressed at different levels in the trigeminal ganglion and that their respective *Sema3A* and *Sema3F* ligands are expressed in distinct patterns in ocular tissues, we analyzed *Npn1^Sema−/−^* and *Npn2^−/−^* mutant embryos for development of corneal innervation. Previous studies of *Npn1^Sema−/−^* mutant embryos have shown premature innervation of the limbs [40] and aberrant projection of axons in the ear [43]. Similarly, we show that axons are misguided in *Npn1^Sema−/−^* mutants resulting in precocious, disorganized, and increased innervation of the corneal stroma. *Npn1^Sema−/−^* mutant corneas were innervated by E12.5, and the patterning and the length of axon projections into the stromal quadrants was disorganized compared to wild type littermates [9]. Since *Sema3A* is expressed in the lens epithelium in close proximity to the corneal stroma, it is likely that Npn1/Sema3A signaling guides sensory axons during stromal innervation. In addition, we found that in *Npn1^Sema−/−^* mutants, the corneal epithelium was prematurely innervated at E15.5, a day earlier than wild type littermates. The corneal innervation defects are probably due to early projection of nerve bundles into the stroma because unlike chick, there is no pause in this process during normal development of the mouse cornea. When lens-derived Sema3A signaling is blocked, neural crest cell progenitors fail to migrate appropriately resulting in abnormal development of the cornea [22]. While there is a possibility that malformation of cornea may contribute to the corneal innervation defects in chick, this does not appear to be the case in mouse. There were no apparent defects in neural crest cell migration during mouse cornea development and all layers were formed normally in the *Npn1^Sema−/−^* mutants (data not shown). Interestingly, unlike chick where *Npn1* is expressed early in the periocular region [21], [22], it is not expressed in the mouse during active migration into the presumptive cornea region between E10.5–E11.5 (data not shown). This may explain the insensitivity of mouse periocular neural crest cells to lens-derived Sema3A signaling and the differences between chick and mouse cornea development.

In contrast to *Npn1^Sema−/−^*, innervation of the corneal stroma was not affected in *Npn2^−/−^* mutants. This is not surprising since *Npn2* and *Sema3F* are expressed at low levels in the trigeminal ganglion and corneal stroma during this time (Fig. 1F–1J and H′–j′). However, similar to *Npn1^Sema−/−^*, the epithelium is prematurely innervated at E15.5 in *Npn2^−/−^* mutant corneas. At this time *Sema3F* is strongly expressed in the corneal epithelium, therefore its premature innervation could be due to lack of response to Sema3F signaling in this layer despite basal levels of *Npn2* expression in the trigeminal ganglion. Although *Npn1^Sema−/−^* and *Npn2^−/−^* mutant embryos have defective trigeminal ganglia that are severely defasciculated at the sites of their targets [18], [37], innervation of the corneal stroma is only affected in *Npn1^Sema−/−^*. This suggests that further signaling at the target site is required for proper innervation since in the absence of Sema3F/Npn2 signaling, Sema3A/Npn1 signaling is sufficient to guide axons into the corneal stroma. However, we cannot rule out defasciculation of the trigeminal ganglion in *Npn1^Sema−/−^* as a contributing factor to the ectopic nerve bundles in the cornea periphery and increased stromal innervation.

Finally, we analyzed *Npn1^Sema−/−^*;*Npn2^−/−^* double mutants for corneal innervation. Defects in gangliogenesis of the trigeminal ganglion are increased in *Npn1^Sema−/−^*;*Npn2^−/−^* double mutants compared with single mutants [37]. Our results show that the defects in mouse corneal innervation are exacerbated in the absence of Sema/Npn signaling. Both the stroma and epithelium were prematurely innervated by E12.5 in *Npn1^Sema−/−^*;*Npn2^−/−^* double mutants. In addition, double mutant eyes showed ectopic nerve projections between the lens and optic cup. Although Sema3A^−/−^ mutants show ectopic innervation in the lens vesicle [15], this was not evident in *Npn1^Sema−/−^* or *Npn2^−/−^* single mutants. These data further suggest that Sema3A/Npn1 and Sema3F/Npn2 signaling act synergistically to guide axon projections during corneal innervation.

In summary, we have examined the roles of Sema3A/Npn1 and Sema3F/Npn2 signaling during mouse corneal innervation (Fig. 6). We find that Sema3A/Npn1 is required for the innervation of the corneal stroma and epithelium, whereas Sema3F/Npn2 is only required for epithelial innervation. The distinctive functions in corneal innervation of Sema3A/Npn1 and Sema3F/Npn2 are further confirmed by the increase of defective axon projections in the absence of Sema/Npn signaling. Since adult corneal nerves are repulsed when Sema3A is ectopically added to the cornea [44], our results may provide insight into the mechanisms that control regeneration of corneal nerves in wounded and diseased corneas.
